# Supporting informed decision making when clinical evidence and conventional wisdom collide: papers developed from the Eisenberg Center Conference Series 2012

**DOI:** 10.1186/1472-6947-13-S3-S1

**Published:** 2013-12-06

**Authors:** Robert J Volk, Richard Street, Quentin Smith, Michael Fordis

**Affiliations:** 1 John M. Eisenberg Center for Clinical Decisions and Communications Science at Baylor College of Medicine, Houston, Texas - 77030, USA; 2 Department of General Internal Medicine, The University of Texas MD Anderson Cancer Center, Houston, Texas - 77030, USA; 3 Department of Communication, Texas A&M University, College Station, Texas - 77843, USA; 4 Center for Collaborative and Interactive Technologies, Baylor College of Medicine, Houston, Texas - 77030, USA

## AHRQ involvement in exploring issues that impact clinical decision making

The AHRQ Effective Health Care (EHC) Program was created in 2003 under the legislative provisions of the Medicare Prescription Drug, Improvement, and Modernization Act (MMA). The EHC Program supports individual researchers, research centers, and academic organizations working together with AHRQ to produce effectiveness and comparative effectiveness research for various audiences (http://www.effectivehealthcare.ahrq.gov). The EHC Program does this through: 1) reviewing and synthesizing published and unpublished scientific evidence; 2) generating new scientific evidence and analytic tools; and 3) compiling research findings that are synthesized and/or generated and translating these materials into useful formats for clinicians, consumers, and policymakers. Much of the work focusing on translation and dissemination of research findings is done through the John M. Eisenberg Center for Clinical Decisions and Communications Science (the Eisenberg Center), a specialized Center within the EHC Program charged with working in concert with other EHC Program components to organize research results into summaries and other tools that are useful to clinicians, healthcare policy makers, and patients. An important function of the Eisenberg Center involves planning and implementing the Eisenberg Conference Series. This series brings together experts in health communication, health literacy, shared decisionmaking, and related fields to produce white papers (and stakeholder commentaries) that explore developments and advances in the fields of clinical decisionmaking and health communication. The papers from the 2012 Conference Series are assembled here as a supplement in *BMC Medical Informatics and Decision Making*.

## Conventional wisdom and evidence in clinical decision making as a theme

While reference to “conventional wisdom” dates into the mid-nineteenth century, the term was popularized by the economist John Kenneth Galbraith, who stated in his book, *The Affluent Society*, published originally in 1958 and re-issued periodically, including the 40^th^ anniversary version cited here,

“It will be convenient to have a name for the ideas which are esteemed at any time for their acceptability, and it should be a term that emphasizes this predictability. I shall refer to these ideas henceforth as the conventional wisdom” ([1] p.8)

The concept proved useful in describing resistance to new thinking in academic economics.

Resistance to new ideas is so pervasive that conventional wisdom has been used to distinguish a new journal from its peers to potential authors and readers. Krumholz in an editorial for *Circulation: Cardiovascular Quality and Outcomes* opens in an editorial titled Questioning Conventional Wisdom that “In science, what seems obvious may not be true, and what is accepted as conventional wisdom, may sometimes be based on flawed assumptions” ([2] p.59). In closing he emphasizes to aspiring authors interested in publishing results of studies that may counter current practice or mainstream thinking that that journal will welcome such submissions—“If your work is done well and your conclusions follow your arguments and data, you will not be disqualified because your conclusions are at odds with conventional wisdom” ([2] p.60)

Perhaps even more challenging is the recognition of tensions arising from popular notions held by patients. Schover contrasts popular beliefs presented in the media with evidence regarding the true story of patient outcomes and quality of life in breast cancer survivorship [3].

For consumers, messages in the media may represent a major source of healthcare information. Friedman and colleagues explored consumer and primary care provider beliefs and communication practices relevant to the subject of maintaining cognitive functioning as an individual ages [4]. They reported that much of what consumers believed about the role of different factors (e.g., diet, exercise, extant disease) in maintaining cognitive functions over time was not informed by information received from clinical professionals—only 7.8% had received information on this issue from health providers—but rather by information obtained from television sources (50.1%); magazines (44.1%) and newspapers (33.7%) [4].

Messages in the media can lead to changes in behavior for which there is no evidentiary basis. Strawbridge in the Harvard Public Health blog discussed the increasing adoption of gluten-free eating habits by individuals with no clinical intolerance to glutens, based on little more than testimonials in the media [5]. She noted that a growing number of people were adopting gluten-free eating habits even though Dr. Daniel Leffler, the director of clinical research at the Celiac Center at Beth Israel Deaconess Medical Center, offered that those without gluten sensitivity “…will derive no significant benefit from the practice. They’ll simply waste their money, because these products are expensive” [5]. Apart from the higher costs associated with a gluten-free diet, such a diet can set people up for nutritional deficiencies (e.g., insufficient vitamin B, the chief source of which for many in the U.S. is fortified bread products; inadequate levels of dietary fiber, which is also available through breads and grain cereals) [5]. A shifting conventional wisdom toward a dietary regimen that primarily benefits people with specific food sensitivities can have significant consequences for others who buy into the “wisdom” but who lack the clinical findings that make the dietary choice a wise one for those who suffer from gluten sensitivity.

A Capstrat Public Policy Poll conducted in 2010 [6] reveals the complexity of the information source landscape facing consumers and healthcare providers. The poll indicated that, while doctors were considered to be the “most influential” sources of health information by respondents to the poll (44% of respondents ranked doctors as most influential), Google searches were the cited as the next most influential (22% of respondents made this selection). However, other sources of information selected by poll respondents included advocacy groups (8%), talks with nurses (8%), online forums (4%), family and friends (2%), pharmacists (2%), and insurance Web sites (1%). The polling results also highlighted differences in the reliability and/or influence levels of specific information sources among different subsets of the respondent populations (e.g., higher proportions of African Americans versus the general population reported Google search results as reliable and influential sources of health information; a higher proportion of women versus men reported that information from nurses was reliable, and women were more likely to seek out friends and family to obtain health information than were men).

Furthermore, “conventional wisdom” may mean different things to different people. Apart from being influenced by belief systems, conventional wisdom may also reflect the processes, heuristics, or rules of thumb used in decision making so aptly investigated and recently summarized by Kahneman in *Thinking*, *Fast and Slow *[7].

## The 2012 Eisenberg Conference Series meeting and resulting papers

In fall of 2012, on behalf of the Agency for Healthcare Research and Quality, the John M. Eisenberg Centers for Clinical Decisions and Communications Science convened a diverse group of experts to describe their experiences and perspectives and to explore issues around *Supporting Informed Decision Making When Clinical Evidence and Conventional Wisdom Collide*. Three separate sessions were convened with three expert presenters providing relevant information in each of the first two sessions and two expert presenters in the third session, for a total of eight presentations. Each session was followed by a discussion period involving all of the presenters, invited discussants with expertise in the topical area, and AHRQ staff and faculty and staff of the Eisenberg Center at Baylor College of Medicine. The sessions were titled I. *Addressing Tensions When Conventional Wisdom*, *Clinical Policy/Practice*, *and Evidence-based Care Collide;* II. *Addressing Tensions When Popular Media and Evidence-based Care Collide;* and III. *Addressing Tensions When Social/Family Support and Evidence-based Care Collide.* From the eight live presentations, six papers were developed for publication, one from Session I (Santa); three from Session II (Schwitzer, Jensen, and Kravitz); and two from Session III (Siminoff and Sanders Thompson). These six papers are presented in this special issue of *BMC Medical Informatics and Decision Making*. The accompanying presentations for all eight speakers are available on AHRQ’s Effective Health Care Program Web site at http://effectivehealthcare.ahrq.gov/index.cfm/who-is-involved-in-the-effective-health-care-program1/about-the-eisenberg-center/eisenberg-center-conference-series-2012/.

It is worth noting that these papers represent a mix of styles and presentation formats that reflect the involvement of authors from different sectors of the media, communication science, and academic medicine. When reading these papers, the differences in styles will be obvious; and we avoided the temptation to bring uniformity to the pieces which reflect the diverse disciplines and backgrounds of the authors. The authors provided perspective pieces that include a mix of research reviews, descriptions of experiences, analyses, and approaches to the challenges of addressing tensions arising from conventional wisdom that may be at odds with evidence, and recommendations for the future.

The articles developed from presentations in each of the three sessions are described in brief below, with the full papers presented as separate articles in this special issue of *BMC Medical Informatics and Decision Making*.

**■** John S. Santa, MD, MPH, director of the *Consumer Reports* Health Ratings Center, presented “Communicating Information About, ‘What Not to Do’ to Consumers.” After providing a brief overview of the history of *Consumer Reports* that extends back 77 years, Santa summarized their decision to establish health care as a “franchise” within the organization in response to their recognition that, with a growing proportion of America’s wealth devoted to health services, consumers were vulnerable to the same kinds advertising and promotional techniques around health care issues that *Consumer Reports* has seen used to influence the purchase of goods and services in other sectors. *Consumer Reports*, Santa writes, is uniquely qualified for such a role due to its “independence from industry, commitment to the best data possible, iconic presentation skills, and a distribution network of tens of millions of savvy consumers.”

Santa offers the example of screening tests for cardiovascular disease, and describes the common usage of diagnostic and screening tests rated by the U.S. Preventive Services Task Force (USPSTF) based on benefits, risks, and costs, with some of the more commonly performed tests (e.g., electrocardiogram and stress test) identified as having no benefit and moderate levels of risk and cost. He goes on to describe how these findings and other experiences with sharing clinical evidence with consumers prompted *Consumer Reports* to enter into a partnership with the American College of Physicians, the American Board of Internal Medicine Foundation, and other professional societies to establish the *Choosing Wisely®* campaign to help inform consumers about ways to obtain high-value care. *Choosing Wisely®* was launched in April 2012; and based on follow-up data, the partnership estimates that by February 2013 approximately 80 million consumers had seen content about one or more of the *Choosing Wisely®* topics. In closing Santa cites the difficulties of countering the conventional wisdom that “more is better” or “more expensive is better” and he emphasizes the importance of the “rightness” of the message and the trustworthiness of the source(s) to ensure that information about the benefits and harms of medical products are understood and remembered by consumers.

**■** Gary Schwitzer is publisher of and writer for the http://HealthNewsReview.org, a St. Paul Minnesota online information service committed to improving “the public dialogue about health care by helping consumers critically analyze claims about health care interventions and by promoting the principles of shared decision-making reinforced by accurate, balanced and complete information about the tradeoffs involved in health care decisions” [8]. In his examination of how stories published through various media outlets may influence conventional wisdom concerning medical interventions, Schwitzer describes how http://HealthNewsReview.org has analyzed health-related news stories using specific criteria related to an intervention’s benefits, harms, and costs; comparisons to alternative options; sources of information; evidence quality and other measures.

The results of these analyses point to a barrage of stories which, in Schwitzer’s words, contribute to a “daily drumbeat of unbalanced messages.” Among media themes that contribute to lack of balance are: use of relative versus absolute risk reduction data, framing potential benefits in the most positive light; overreliance on anecdotal information without discussing countervailing information (e.g., trial drop-outs, compliance problems); and the strength and scope of information sources (i.e., heavy reliance on single information sources). Using the breast cancer screening controversy that emerged from the 2009 changes in USPSTF screening recommendations as an example, Schwitzer highlights the important role that media can play in either contributing to or detracting from constructive public debate about health issues.

Schwitzer also identifies possible actions that might be taken by government, private organizations, not-for-profit organizations, and others to improve the quality of scientific information and evidence shared with the public.

**■** Jakob D. Jensen, PhD, and colleagues, Drs. Krakow, John, and Liu (all with the Department of Communication at the University of Utah), also focused their discussion on the role of media in informing the public about clinical practice. Like Schwitzer, these authors used the breast cancer controversy following the USPSTF changes in screening recommendations to illustrate their points. However, rather than focusing on imbalance in media reporting on the changes, Jensen and colleagues explored issues regarding the means via which scientific findings are communicated to the public and the ways in which such concepts may be misconstrued either by the media or the public. Scientific information may get “streamlined” as it moves through communication channels, removing complexity and uncertainty and altering messages. The authors offer examples of how media outlets may alter message content to heighten the impact of their reporting with the goal of attracting more readers. The need to reconsider how uncertainty, in particular, is conveyed to target audiences, including through use of visual depictions, is explored, and increasing applications of interactive media in conveying uncertainty is cited as a promising strategy. Jensen concludes with cautions regarding trends toward oversimplification of scientific findings, particularly as a strategy for accommodating audiences with general or health literacy deficiencies and emphasizes the need for communicating indicators of uncertainty in health-related recommendations to ensure long-term coherence in messaging.

**■** Richard Kravitz, MD, MSPH, professor of internal medicine at the University of California (UC) at Davis and his co-author, Robert Bell, PhD, from the Departments of Communication and Public Health Sciences at UC-Davis, approached the issue of media in health communication from the perspective of how media affects communications between clinicians and patients regarding the prescription and use of medications. Kravitz reviews the literature to examine four questions: What information are patients exposed to, and are they paying attention? Is information that patients receive credible and accurate? When patients ask for a prescription, what do they want and need? And, can physicians reconcile what patients hear, want, and need?

Kravitz and his colleague explore issues around direct-to-consumer advertising (DTCA) and the effects that the Internet has had in expanding access to information on pharmaceutical products. They note the irony in the fact that, although consumers may be “swimming in a sea of health-related information,” they may still feel unprepared to participate in health care decisions. In response, the authors discuss the challenges consumers face in determining which information sources to trust and how consumers may act upon the messages that they receive from the media relevant to their conversations with their clinicians. The question about patients getting what they want and what they need is especially thorny, as the authors suggest. While patient “wants” are often based on misunderstandings and/or concerns arising from personal or family history, the authors clearly delineate three things that patients need from their doctors: valid evidence, clinical discernment, and a healing relationship. They stress the important role of the clinician as a listener who takes the time to probe factors (e.g., fear of disease progression, death or disability) that may have powerful influences on expressed wants or needs for medications that may not benefit the patient, and may in fact put them at undue risk. In answering the fourth question related to reconciliation of patient wants and needs, Kravitz and Bell suggest changes in policy around both the dissemination of pharmaceutical product information and the preparation of clinicians to discern better what a patient may be expressing when he or she comes into the practice demanding a product he or she has heard about through the media.

**■** Laura Siminoff, PhD, chairman and professor of the Department of Social and Behavioral Health in the School of Medicine at Virginia Commonwealth University authored a paper that focuses on incorporating patient and family preferences into evidence-based medicine.

Following a brief discussion about how patient-centered care differs from traditional care (i.e., tempering clinical evidence with information that acknowledges the importance of the ways in which a patient experiences illness), Siminoff offers examples of ways in which concerns of clinicians may differ from concerns of patients to reflect upon the critical role of patient values and preferences in clinical decision making.

Introducing the influence and role of family and caregivers into the decision making process, Siminoff describes two research initiatives—one involving treatment seeking behaviors of patients with colorectal cancer (CRC), and the other involving treatment decisions by patients diagnosed with lung cancer—to better understand how family and supporters influence medical decision making. What emerges from the two studies is confirmation of the important role played by friends and family in decisions that patients make concerning the actions that they will take in: a) following up on preliminary diagnostic results; and b) determining the course of treatment once a diagnosis is made. Examples of the degree of involvement of family members in health decisions, including examples that suggest that such involvement can sometimes be perceived as stifling discussion between patients and clinicians, are offered. In the conclusions, Siminoff offers the important insight that illness is “… not just a biological process, but also a social process.” She concludes with recommendations to clinicians regarding a series of steps that they might pursue in establishing treatment plans that are patient-centered and that acknowledge the important roles often played by family and friends in the medical decision making process.

**■** Vetta Sanders Thompson, associate professor in the George Warren Brown School of Social Work at Washington University, explores the effects of communication approaches and also an individual’s social network on her health care decision making processes. Thompson reviews findings from research on studies of health care disparities related to race, ethnicity, and socioeconomic status. Thompson points out differences in the ways in which people from differing cultural backgrounds tend to seek health information. She cites data supporting the thesis that, in many cases, the sources of information available to support understanding of health issues and facilitate health decision making are poorly matched to the educational levels, language skills and information needs of patients confronting a health issue. The mismatches are often particularly evident in addressing the needs of people from low income, less well-educated groups, including older persons from racial and ethnic minority groups.

Such problems may be exacerbated in cases where the information being shared relies substantially on statistical data, as is often the case in explaining risk for an occurrence and why understanding of the degree of risk may be very important in making a good health care decision. In suggesting strategies for communicating more effectively with health care consumers who have difficulty with numeracy issues, including understanding statistical concepts that are important in determining risk levels, Thompson explores the potential of using anecdotes and personal testimonials in helping people to grasp the importance of evidence, internalize into their decision processes, and share it with other members of their social network involved in health care decision making.

She suggests that by combining information presented in narrative formats (e.g., anecdotes, testimonials) with statistical data in discussing the potential benefits and risks of harm associated with clinical interventions, the clinician can tap into the important resource that is represented by the social norms of a group or culture, capitalize on attitudes shaped by community interactions, and draw upon the trust that exists within social networks developed over time.

In her conclusions, Thompson notes the need for more research and greater understanding of how emerging strategies that integrate social narrative with quantitative data (e.g., social math) can be effectively integrated into “clinical conversations” that promote better understanding by all parties in the information exchange. She also suggests that research into appropriate and effective ways of bringing members of social networks into the decision process in constructive ways and in ways that are acceptable to all parties (i.e., the patient, family and friends, the clinician) is needed, as is work on ways to leverage the potential of tools like the Internet.

## Closing statement

The authors of the six papers in this supplement provide insights into experiences, challenges and approaches relevant to communication in settings where evidence may collide with conventional wisdom. Santa provided insights from national campaigns that encounter resistance from a public trained to believe that ‘more is better’ or ‘newer is better’. Schwitzer, Kravitz, and Jensen each explored aspects of communication of evidence in settings where messages may be filtered through intermediaries such as the press or Internet authors. And Siminoff and Sanders Thompson consider the influence on patient decision making of conventional wisdom arising from family, peers, and social networks.

Across the papers, several themes emerge which include: (1) the importance of understanding the source, content, and nature of conventional wisdom that may be a source of resistance; (2) clarifying (particularly in clinician-patient communications) other issues, concerns, and needs of patients that may influence or be disguised in expressions of ‘conventional wisdom’; (3) providing clear and understandable information and its limitations (uncertainties) in forms and formats suitable for audiences and their support networks; (4) confirming understanding of communications; and (5) finally readiness to improve, on a continuing basis, efforts to advance effective communication of evidence.

## Competing interests

The authors declare that they have no competing interests.

## Declarations and disclaimer

The Eisenberg Conference Series 2012, *Supporting Informed Decision Making When Clinical Evidence and Conventional Wisdom Collide*, was conducted in Rockville, Maryland, by the John M. Eisenberg Center for Clinical Decisions and Communications Science, Baylor College of Medicine, Houston, Texas, for the Agency for Healthcare Research and Quality under Contract No. HHSA 290-2008-10015-C. Publication costs for this supplement were funded by this contract. The author of this article is responsible for its content. No statement may be construed as the official position of the Agency for Healthcare Research and Quality and of the U.S. Department of Health and Human Services.

This article has been published as part of *BMC Medical Informatics and Decision Making* Volume 13 Supplement 3, 2013: Articles from the Eisenberg Center Conference Series 2012: Supporting informed decision making when clinical evidence and conventional wisdom collide. The full contents of the supplement are available online at http://www.biomedcentral.com/bmcmedinformdecismak/supplements/13/S3.
